# Environmental drivers of the occurrence and abundance of the Irukandji jellyfish (*Carukia barnesi*)

**DOI:** 10.1371/journal.pone.0272359

**Published:** 2022-08-04

**Authors:** Olivia C. Rowley, Robert Courtney, Tobin Northfield, Jamie Seymour

**Affiliations:** 1 Australian Institute of Tropical Health and Medicine, James Cook University, Cairns, QLD, Australia; 2 Tree Fruit Research and Extension Center Washington State University, Wenatchee, Washington, United States of America; Università del Salento, ITALY

## Abstract

Understanding the links between species and their environment is critical for species management. This is particularly true for organisms of medical and/or economic significance. The ‘Irukandji’ jellyfish (*Carukia barnesi)* is well known for its small size, cryptic nature, and highly venomous sting. Being the namesake of the Irukandji syndrome, contact with this marine stinger often leads to hospitalization and can be fatal. Consequently, the annual occurrence of this organism is believed to cost the Australian government an estimated $AUD3 billion annually in medical costs and losses for tourism. Despite its economic importance the logistical difficulties related to surveying *C*.*barnesi in situ* has led to a paucity of knowledge regarding its ecology and significantly impeded management strategies to date. In this study, we use six years of direct *C*. *barnesi* capture data to explore patterns pertaining to the annual occurrence and abundance of this species in the nearshore waters of the Cairns coast. We provide novel insights into trends in medusae aggregations and size distribution and primarily focus on the potential role of environmental drivers for annual *C*. *barnesi* occurrence patterns. Using a two-part hurdle model, eight environmental parameters were investigated over four time periods for associations with records of medusa presence and abundance. Final models showed a small amount of variation in medusa presence and abundance patterns could be accounted for by long-term trends pertaining to rainfall and wind direction. However, the assessed environmental parameters could not explain high annual variation or site location effects. Ultimately best-fit models had very low statistical inference power explaining between 16 and 20% of the variance in the data, leaving approximately 80% of all variation in medusa presence and abundance unexplained.

## Introduction

Understanding the link between environmental variables and the temporal distribution and occurrence patterns of a species has long stood as a primary goal for ecological investigations [1–3]. Originating from direct or indirect species identification occurrence data informs presence and abundance estimates and ultimately species prediction [4]. When confronted with core environmental parameters and statistical analysis these data can link an animal’s population dynamics to core environmental drivers [1, 2, 4, 5]. Such ecological insight is invaluable as data regularly forms the foundation for species management decisions aimed at tasks such as identifying habitats vulnerable to range expansion, locating unknown populations of high importance, and also the management of species of significant economic or medical consequences [2, 4].

One such medically important species is *Carukia barnesi*. Commonly known as the Irukandji jellyfish, *C*. *barnesi* is a member of the class Cubozoa (box jellyfish) and is characterized by its small (~30mm), transparent, cube-shaped bell [6–9]. Being the namesake of the ‘Irukandji syndrome’, this marine invertebrate is well known for its highly venomous sting and contact with this marine stinger can result in a suite of symptoms that require immediate medical attention, including nausea, vomiting, and extreme pain [8, 10]. Additionally, on occasion envenomation’s from *C*.*barnesi* can even be fatal [10]. *Carukia barnesi* medusa exhibit marked seasonality in synchrony with the summer monsoonal months occurring in the tropical waters of North-Eastern Australia from November to May each year [7, 8, 11, 12]. During this termed ‘stinger season’ the direct costs associated with treating sting victims is an estimated one to three million dollars per year and, while more difficult to estimate, the loss of revenue for the local tourism industry is substantial [13].

Despite the medical and economic significance of *C*. *barnesi*, biological understanding surrounding the basic ecology of this species is severely limited [7]. This is primarily due to characteristics that make tracking and studying this species logistically difficult, such as their small size, venomous nature, metagenetic lifecycle (alternating between an asexually reproducing sessile polyp and a sexually reproducing free-swimming medusa), and patchy distribution [12]. As a result, while large-scale annual occurrence trends for *C*. *barnesi* are generally well observed, due to their regularity, research into finer scale inter-annual occurrence and abundance patterns of medusa and their underlying mechanisms are rare. Consequently, the coarse nature of the current phenological understanding of *C*. *barnesi* occurrence leads to scenarios where health officials can block off large parts of the year when stings are possible but cannot give reliable advice for a particular week or day early or late in the stinger season.

Currently, research on these smaller-scale inter-annual jellyfish occurrence patterns are dominated by work on related Scyphozoan species [12, 14]. This trend is in large part due to the comparatively large diversity and high prevalence of Scyphozoan jellyfish, often occurring in large blooms comprised of thousands of individuals, and the negative impact these blooms have on human activities resulting in occurrences/outbreaks being well documented in long-term historical records [12, 14, 15]. Although some studies highlight the influence of anthropogenetic stressors, on patterns of jellyfish occurrence and abundance [16–18], the importance of physical environmental factors such as currents, wind speed, tide, and temperature are also very apparent [15, 16, 19–21]. While less abundant, studies on occurrence and abundance patterns for Cubozoa do exist with literature on closely related species, such as *Chironex fleckeri*, *Alatina alata*, *Chiropsella bronzei*, and *Carybdea marsupialis*, also suggesting occurrence patterns can be influenced by environmental drivers such as lunar phase, rainfall, freshwater input, sea surface temperature, and salinity [14, 22–25]. However, to date, only two studies document *in situ* occurrence patterns for *C*. *barnesi* [12, 13] and, of these, only one has the Irukandji jellyfish as its main focus [13]. Furthermore, this study relied on hospital sting databases as a source of indirect occurrence records and given envenomations from *C*. *barnesi* are hard to identify (particularly due to the delay of symptom onset and lack of visual identification of envenomation site), and that data such as these are difficult to standardize post hoc [26], there is a need for a long-term direct investigation of annual *C*. *barnesi* occurrence and abundance patterns and their potential environmental drivers [8, 12, 13].

In this study, we utilized the direct capture of *C*. *barnesi* and considered a variety of environmental and temporal parameters as core environmental drivers of the inter-annual occurrence patterns of this organism. We evaluate the strength of these relationships and discuss the capacity of these associations as a basis for management approaches and predictive forecasting tools. Furthermore, by combining six years of data, from targeted animal surveys comprised of direct species identification and animal capture, this paper offers a comprehensive alternative to the traditionally used sting database for occurrence and abundance estimates for this medically important, logistically difficult, organism.

## Methods

### Specimen collection, species identification, and size measurements

**Fig 1:**
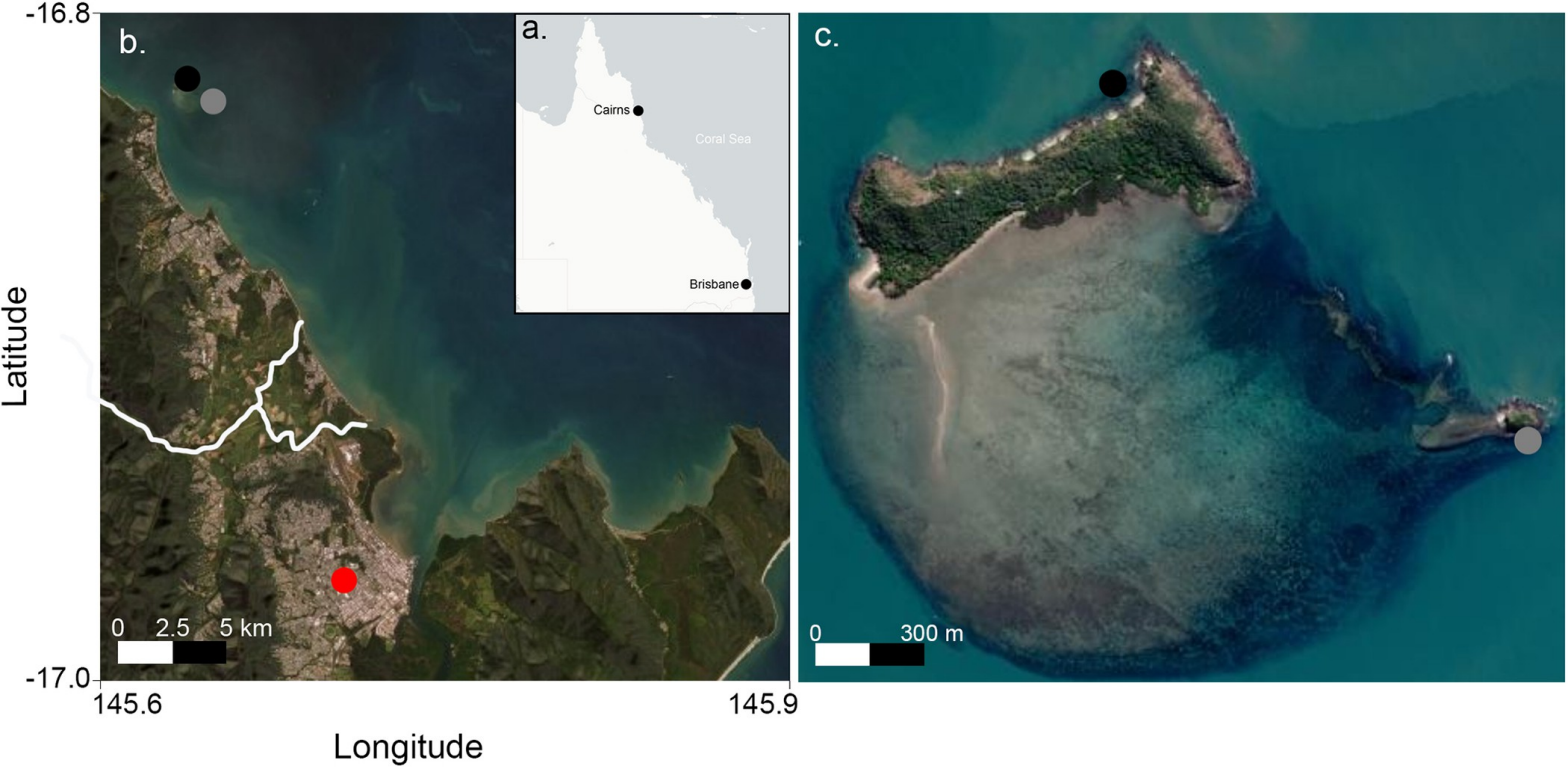
Irukandji sampling sites. a—Map of Queensland Australia (Brisbane and Cairns have been identified as reference points), b—Map of Cairns region and survey sites. Double Island–Black circle, Haycock Island–Grey circle, and Cairns City–Red circle. The Barron River is indicated by a white line. c–Detailed map of sampling sites. Double Island–Black circle and Haycock Island–Grey circle. All images are oriented in a north to south direction. All satellite maps were generated using the leaflet package in R (base map data from OpenStreetMap and OpenStreetMap Foundation. All images are freely available for use under the open database license, licensed as CC BY-SA 2.0).

We collected medusae of *Carukia barnesi* from two nearshore island sites, Double and Haycock Islands (Fig 1), in North Queensland Australia over six stinger seasons (November to May) spanning 2013/14–2018/19. All animal capture occurred in a three-hour window between approximately 1900 and 2200h. To attract medusae, high-powered LED lights (1500 lumen, 650k) were submerged just below the sea surface on each side of a 5.8-meter research vessel. *Carukia barnesi* medusae are photopositive and were identified in the water by boat-based observers. Subsequently, medusae were captured as they approached the lights using a scoop net (1mm mesh size) and transferred into individual 500ml plastic containers. Post capture, specimens were transported to the laboratory where species confirmation was made per the description by Southcott [27]. All animals were collected in accordance with permit numbers G11/34552.1 and G15/37396.1 (Department of Agriculture and Fisheries, Queensland, Australia).

Medusa size was determined as the niche bell width (Nb)—a longitudinal measurement from the center of the Topalian niche to the apex of the bell. Individual size measurements were taken using a dissection microscope and 2mm grid and each animal was recorded to the nearest millimeter.

### Time frame and environmental predictor classification

To investigate short-term and long-term drivers of jellyfish occurrence and abundance, and to allow enough time for physical processes to occur and impact jellyfish presence and abundance, our observed environmental predictors were divided into four categories: T_0_ –day of the survey, T_3_ –data pertaining to the day of survey and previous two days, T_5_ –data pertaining to the day of survey and previous four days, and T_7_ –data pertaining to the day of survey and previous six days. Previously, accounting for processes using time lags such as these have improved jellyfish population estimation [15]. We determined the start and end of each stinger season by rapid changes in jellyfish abundance, and temporal bounds for time period classification were defined on the basis of inflection points and seasonal patterns (such as modal days of consistent rainfall events—S1 Fig).

Environmental predictors were collated from several sources and selected based on historical data availability and significance in previous studies [13–15, 22, 28]. Moon visibility, analyzed as the percent of moon disk illuminated, was matched to historical dates using the lunar illuminate function from the R ‘Lunar’ package [29]. Data on Barron River outflow (mean discharge ML/day) was sourced from the Queensland Government water monitoring information portal and was taken from monitoring station 110016A Koah as this was the closest (approximately 10km) uninterrupted station to the estuary and sampling sites. Windspeed (m/s^-1^), wind direction (°), and rainfall (mm) measurements were obtained from the Australian Bureau of Meteorology (BOM) and recordings were sourced from Cairns airport (situated approximately 16km from sampling sites). Estimates of sea temperatures (°C) were obtained from boat-based recordings and cross-referenced with historical recordings obtained via wave rider buoys sourced from BOM. Due to the lack of available historical data, sea surface temperature was only assessed for the day of survey (T_0_). Aside from cyclical predictors (which were investigated at T_0_ only), all environmental variables were averaged across respective temporal periods. Wind direction and weighted wind direction averages were calculated according to the methods of Grange [30].

### Statistical analysis and model selection

To investigate the potential link between patterns of jellyfish occurrence/abundance and environmental variables a two-part hurdle model was used. Hurdle models are often used in zero-inflated count data with a large number of zeroes, where the full model is a composite of two models: a zero hurdle model describing presence/absence, and a count data model describing variability in counts for non-zero data [31]. This statistical approach has important advantages over conventional count data model approaches, primarily by modeling presence/absence and magnitude/abundance separately, we can accommodate the large number of zero counts observed and also investigate the potential for presence/abundance having differing environmental drivers than variability in abundance. The zero hurdle model focused on the likelihood of *C*. *barnesi* presence/absence (indicated by successful and unsuccessful surveys) given environmental predictors. This analyzed jellyfish presence, denoted as binary factor 1, for individual survey efforts with jellyfish counts greater than zero and absence, denoted as binary factor 0, for efforts when no jellyfish were caught. Data were then analyzed via a logistic regression model. Logistic regression is well-suited for the binary (presence/absence) nature of these data and has been used previously to associate patterns of jellyfish presence with environmental variables [15, 28, 32]. The second part of the hurdle model, the count data model, was aimed at investigating drivers of *C*. *barnesi* abundance patterns when counts were greater than zero. This included a generalized linear model with a zero truncated negative binomial error distribution to account for the lack of zero data (since it was conditional on jellyfish presence) and overdispersion. Overarching annual and site-specific effects on jellyfish presence and abundance were analyzed similarly, using logistic regression for analysis pertaining to presence/absence and negative binomial regression for abundance, and the results of these analyses determined how these two factors would be treated in both part one and two of the final models.

Due to the large number of predictor variables, collinearity, and to avoid overfitting, a model selection process was carried out on both parts of the hurdle model. A single global model was generated that included all predictor variables then, using the dredge function from the ‘MuMin’ package [33], all possible univariate and multivariate predictor combinations were assessed using descriptive measures of goodness of fit (log-likelihood, McFadden’s p^2^, Cox and Snell R^2,^ and Nagelkerke R^2^). Final statistical decisions were made based on Akaike’s Information Criteria (AIC). The final model(s) included only those predictors/predictor combinations that best described the data.

All data were collated and analyzed in R (R Core Team 2017) using the ‘Lme4’ [34], ‘MASS’ [35], and ‘MuMIn’ [33] packages. Relationships between all predictor variables were investigated using a Pearson correlation test for collinearity violations. Correlation coefficients greater than 0.5 were deemed to violate the assumption of independence and dropped from the final models. All explanatory variables were observed for outliers and/or leverage points. Final measures of model fit and variability explanation were derived from ‘MuMIn’ [33] and the ‘rr2’ package [36].

## Results

### Annual and site-specific patterns in *Carukia barnesi* presence and abundance

*Carukia barnesi* capture records were obtained for a total of 153 trips over six consecutive stinger seasons, spanning November 2013 to January 2019. The number of individual survey trips, in each year’s stinger season, ranged from 10 to 41 in the 2018/2019 and 2017/2018 stinger seasons respectively (Table 1). All trips represented approximately 460h of survey time.

**Table 1:** Annual records of Carukia barnesi medusa capture and abundance.

Season	Recording Period	location	Number of sampling trips	Jellyfish tally	Season total
2013/14	16 November– 05 April	North Double	19	51	115
		Haycock	11	64	
2014/15	04 December– 04 May	North Double	12	18	58
		Haycock	8	40	
2015/16	23 October– 01 March	North Double	13	133	602
		Haycock	12	469	
2016/17	16 December– 22 January	North Double	10	28	44
		Haycock	8	16	
2017/18	23 October– 06 March	North Double	33	69	81
		Haycock	17	12	
2018/19	29 November– 07 January	North Double	7	30	65
		Haycock	3	35	

Table includes information on stinger season length (period between the first and last sampling effort of the stinger season), sampling location, and associated catch tallies (both an overall stinger season total and also split by site). All sampling occurred between late October and early May as this encompasses the Northern Queensland ‘stinger season’ when medusae are present in the waters of the Cairns Coast. The number of individual medusa sampling trips vary each year due to resource availability and site accessibility.

**Fig 2:**
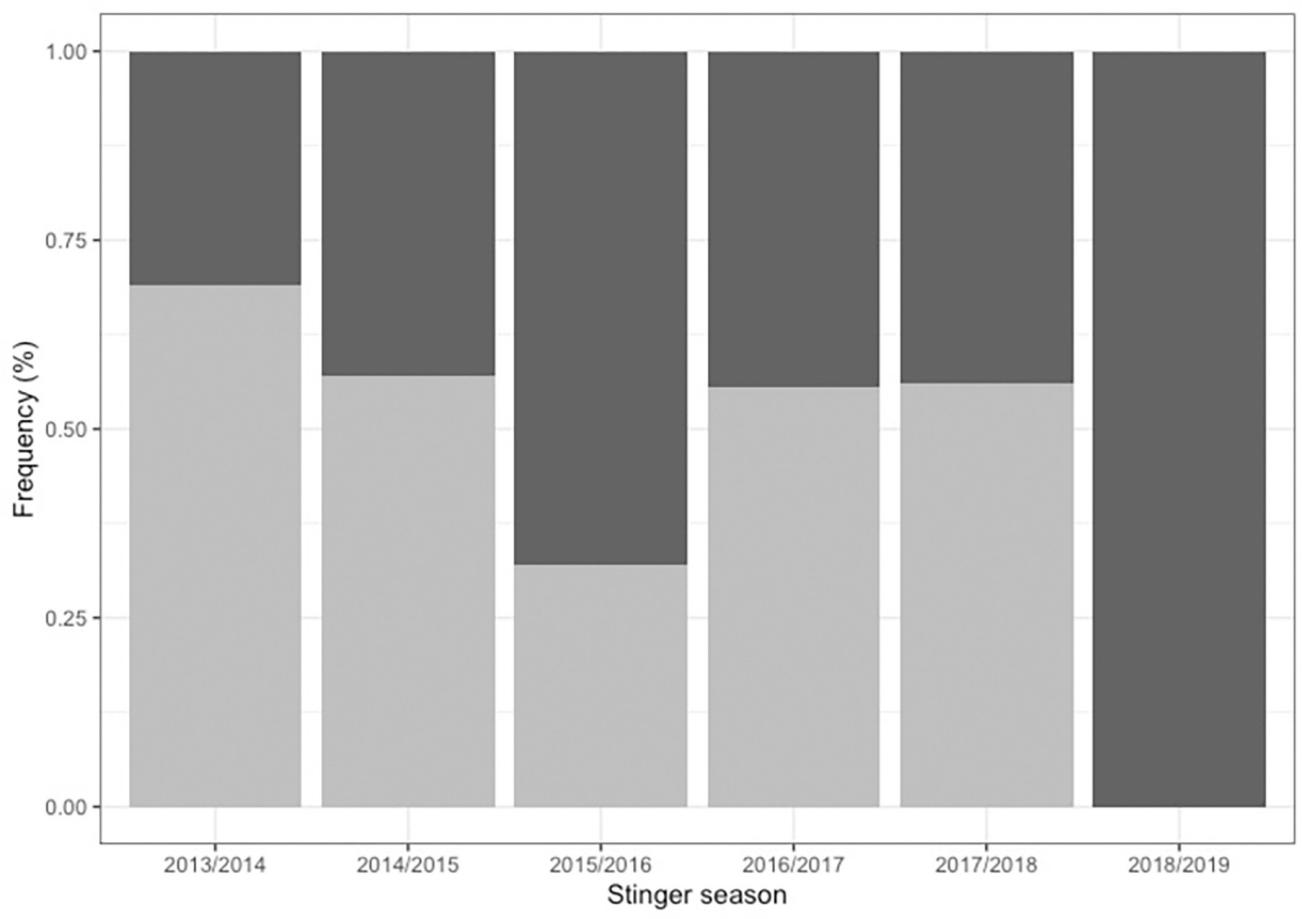
Site and stinger season vs proportion of *C*. *barnesi* presence. Bars represent the proportion of total survey trips where jellyfish were either present and caught (dark grey) or absent (light grey) across the six surveyed stinger seasons. Jellyfish were collected by boat from nearshore Islands via attraction to lights and collected using a scoop net.

Animals were captured on 49% of all survey trips and aside from the 2018/2019 stinger season, which saw only successful survey trips (animals captured), all stinger seasons had both successful and unsuccessful survey efforts (Fig 2). There were significant differences in the relative proportions of total trips for which *C*. *barnesi* were present (and caught) or absent across stinger seasons (F_1,149_ = 4.511, P = 0.035) and post-hoc analysis revealed this difference driven by the 2018/2019 stinger season alone. As a result, the 2018/2019 stinger season was removed from the first part of the hurdle model. No statistical difference was identified in *C*. *barnesi* presence or absence between sites (F_1,149_ = 0.030, P>0.05) and there was no statistical evidence of an interaction effect (F_1,149_ = 0.074, P>0.05).

**Fig 3:**
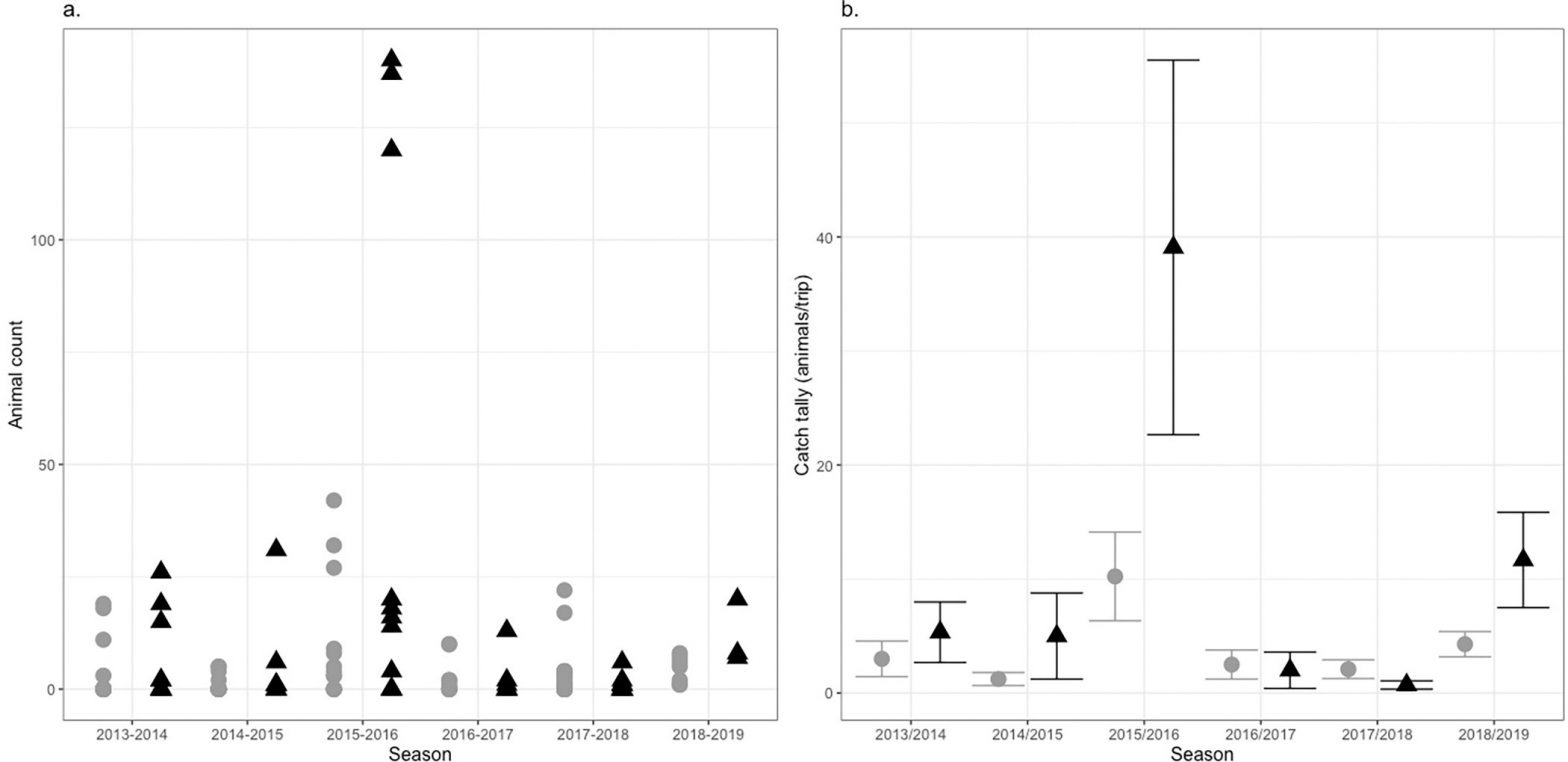
*C*. *barnesi* abundance vs stinger season and site. a—Points represent individual animal counts from singular survey trips. b—Points represent the mean number of animals caught per trip across surveyed stinger seasons (± SE). Data are zero truncated and we only present trips where jellyfish were caught to represent the count data model in the hurdle analysis. All data is grouped by stinger season and site. Points associated with surveys at Double Island are represented by grey circles and Haycock Island surveys are shown using black triangles.

Over the six consecutive stinger seasons 960 individual *C*. *barnesi* medusa were captured between the two sampling sites (Fig 1). The stinger season with the largest catch tally occurred in 2015/2016 when a total of 140 individuals were captured at Haycock island in a given three-hour sampling period. Similar numbers of animals (120 and 137) were caught at the same site in the three days prior (Fig 3A), with significant differences in both site (F_1,72_ = 5.513, P<0.001) and stinger season (F_1,72_ = 1.449, P<0.001) for the number of animals captured. Post-hoc analysis revealed this to be driven by the above-mentioned three survey days. As a result, stinger season and site were included as factors in the final statistical model pertaining to jellyfish abundance and a binomial error distribution (logit link function) reduced the influence of these extreme values on final model results.

Finally, upon adjustment for the total number of trips made per stinger season, and when considered in absence of the 2015/16 stinger season ‘big catch days’, annual catch rates were very consistent. Catch rates continuously fell between 2 and 15 animals per survey trip (Fig 3B) and stinger season averages were 3.8, 2.9, 2.4, 1.6, and 6.5 individuals per sampling trip from the 2013/2014 to 2018/2019 stinger seasons respectively.

### Medusa size-frequency

**Fig 4:**
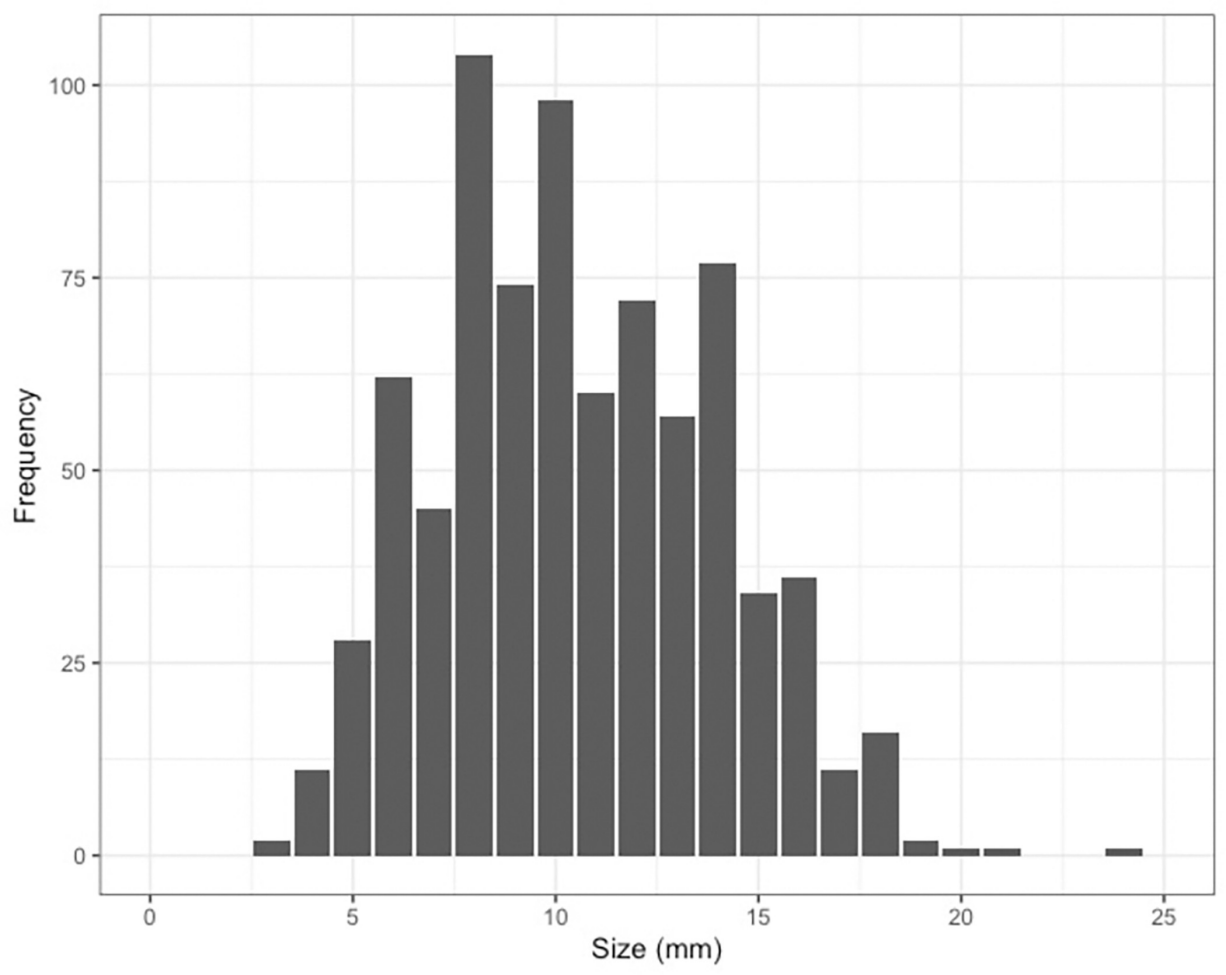
Size frequency distribution of *Carukia barnesi* captured in all sampling locations across all stinger seasons. Size of individuals is measured as niche bell width (mm).

The bell width for *Carukia barnesi* medusa, collected at Double and Haycock Islands between 2013 and 2019, ranged between 3 and 24 mm. The size frequencies of 91% of all jellyfish captured fell between 6 and 16 mm with a peak in abundance at approximately 8mm (Fig 4). Individuals’ grater and less than this size class made up 4% and 5% of observations respectively. No jellyfish were observed less than 3 mm indicating an absence of the smallest size class of *C*. *barnesi* medusa in this study (0.5–3mm).

### Spatio-temporal patterns of environmental parameters

All observed environmental parameters followed a pattern characteristic of Far North Queensland’s tropical wet season. Sea surface temperature, for the analyzed catch days, ranged between 24.6 (January 2018) and 33.5°C (January 2017) with an average sea temperature of 29.4°C.

Rates of rainfall (mm/h) and river outflow (MgL) had a distinct seasonal pattern with the majority of annual rainfall observed through the summer ‘wet’ months and peaking between February and April in the latter half of each stinger season. Mean rainfall (mm/day) and outflow (MgL/day) displayed significant levels of collinearity with correlation coefficients >0.5 (S2A Fig). Thus, to avoid collinearity measures of riverine outflow were removed as a candidate for the final global model on the basis of AIC (S1 & S2 Tables). Analysis of medusa presence/abundance and outflow (T_0_-T_7_) has been included in S1 and S2 Tables for comparison. Consistent days of rainfall had a modal peak at five and seven days representing 8.19 and 5.26 percent of total rainfall days.

All wind directions originated from between 0 and 180°. Given the season, location of sampling sites, and time of data collection this was to be expected. Because the data were bounded by 180° we were able to analyze the data as one continuous linear variable rather than split data into vectors or categorical bins. Wind speed variability over the sampling windows was low, ranging between 3.38 and 8.94 m/s^-1^, and as result, there were a strong levels of co-variance between weighted and non-weighted wind directions (S2B Fig). Consequently, weighted wind direction was exclusively used in the global model, improving AIC values and real-world applicability over non-weighted wind direction. Univariate analysis of medusa presence/abundance vs wind speed (T_0_-T_7_), alongside measures of non-weighted wind direction (T_0_-T_7_), have been included in S1 and S2 Tables for comparison.

### Environmental drivers of *Carukia barnesi* presence/absence

Following model selection, the optimal model for the environmental correlates of *Carukia barnesi* presence/absence (binomial logistic regression, AIC = 192.0) included only long-term measures of rainfall (T_7_) and weighted wind direction (T_7w_). All other environmental predictors, including measures of sea surface temperature, tidal range, and moon phase (alongside the remaining temporal divisions of wind speed, and rainfall at T_0_) did not have a significant effect on model fit and were eliminated from the final selection. The top three ranking models and their components are reported in S3A Table and the full model output is reported in S4 Table. The final logistic regression model, which included measures of both rainfall and wind direction, provided a better fit to the data than the reduced, univariate, models containing either rainfall (LR test statistic = 21.24, df = 1, *P*<0.001) or wind direction alone (LR test statistic = 9.22, df = 1, *P* = 0.002).

**Fig 5:**
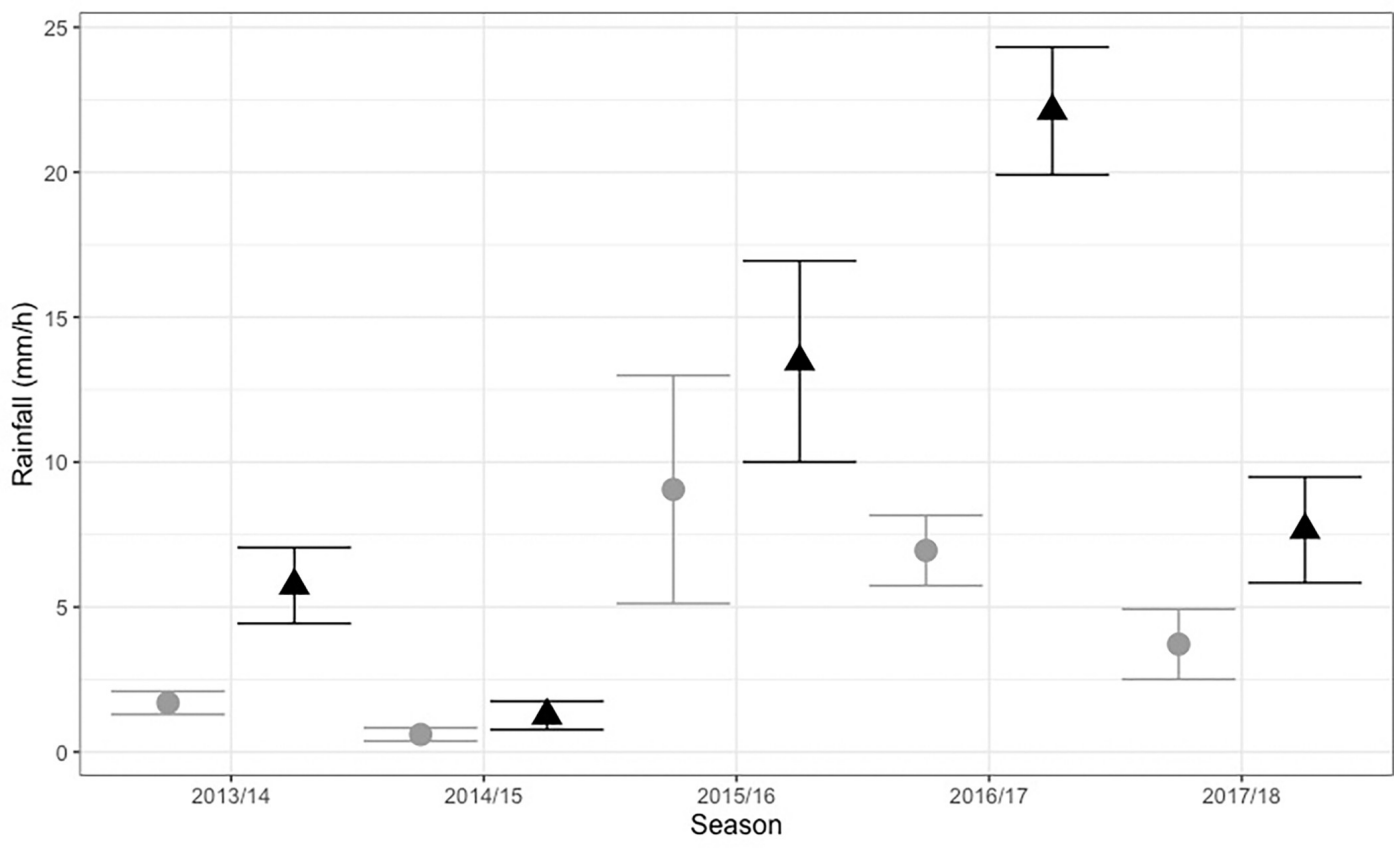
Weekly mean rainfall and *Carukia barnesi* presence. Mean weekly rainfall (T_7_) in mm per day associated with jellyfish absence (grey circle) and presence (black triangle). Plotted data is split by stinger season and each point is representative of the group means ± SE.

The final model showed a positive relationship between the presence of *Carukia barnesi* medusa and rainfall. Survey trips that were associated with higher levels of rainfall, particularly in the week prior to sampling, had significantly increased instances of jellyfish capture when compared to survey trips that did not (F_1,139_ = 19.269, P<0.001). This trend was robust across stinger seasons, with each of the five stinger seasons showing catch days associated with higher levels of rainfall compared to non-catch days (Fig 5). The difference in mean rainfall, between successful and unsuccessful surveys each stinger season, did vary from a minimum of 0.6mm/h in 2014/2015 to a maximum of 22.1 mm/h in 2016/2017. Furthermore, although not selected for the final model, preliminary analyses suggested significant univariate relationships between rainfall and survey success were also established at timeframes T_3_ and T_5_ and this variable became more significant with increasing time periods (see S1 Table for full details). A significant relationship was not identified between jellyfish presence and rainfall at T_0_ (S1 Table for full details).

**Fig 6:**
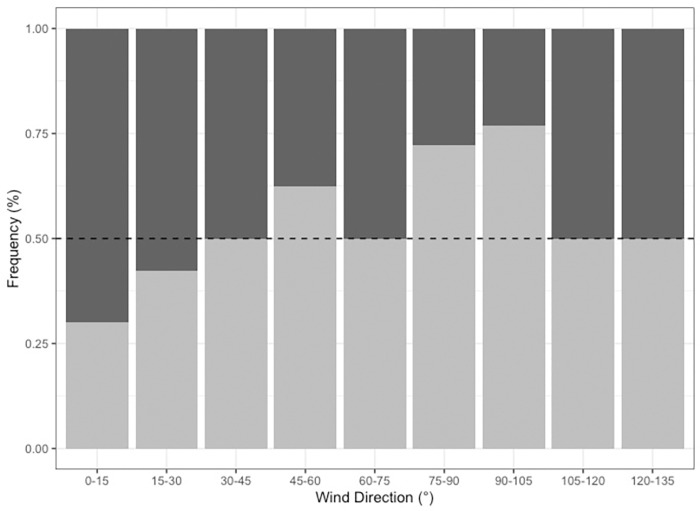
Mean wind direction and *Carukia barnesi* medusa presence/absence. Mean weekly wind direction (T_7_) associated with instances of jellyfish absence (light grey) and presence (dark grey). The graph displays data relating to all sampling trips for both Double and Haycock Island. Count frequency represents the total proportion of all survey efforts for which jellyfish were either present or absent. Data has been grouped into 15° categories for visualization purposes. All data has been weighted for wind speed for averaging purposes using the mathematical method described by Grange [30].

**Fig 7:**
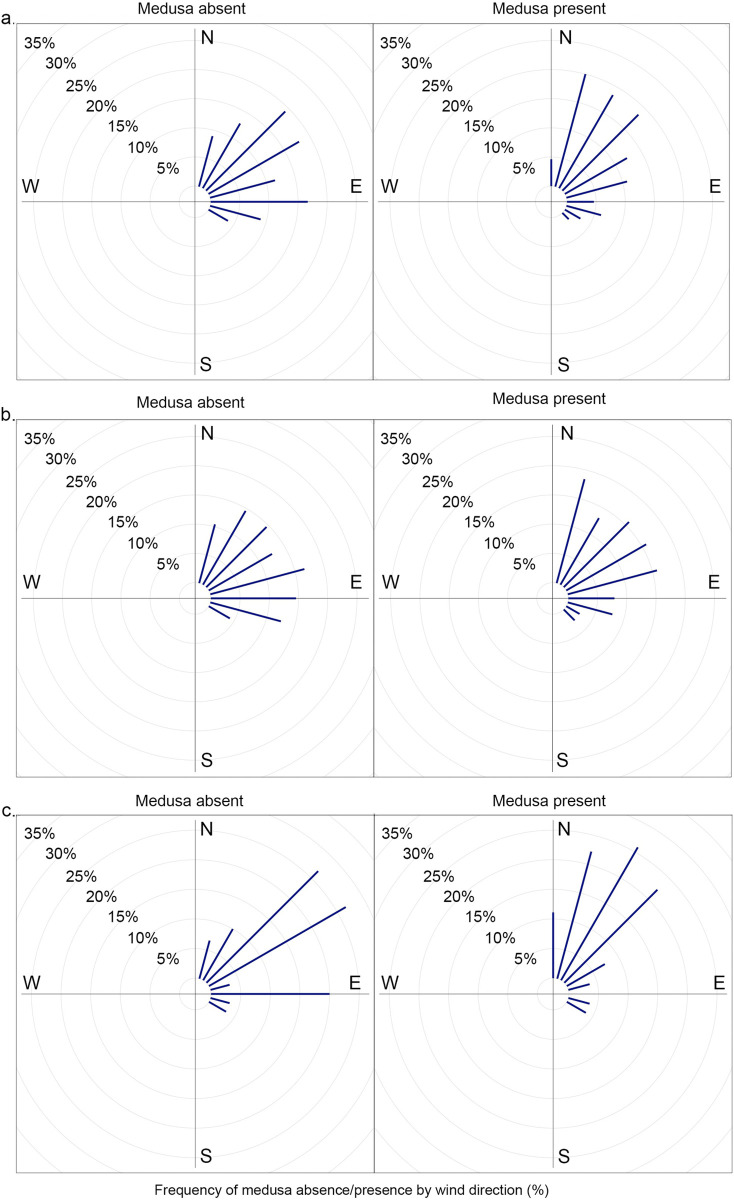
Weekly mean wind direction and *Carukia barnesi* medusa presence/absence. Mean weekly wind direction (T_7_) associated with instances of jellyfish absence (panel one) and presence (panel two). a—shows data relating to all sampling trips (both Double and Haycock islands) and b and c show data relating to each site individually (Double and Haycock islands respectively). The length of bars represents the frequency of counts and the angle of the bar is equivalent to the associated wind direction. Data has been grouped into 15° categories for visualization purposes.

A significant relationship was also established between animal capture and weighted wind direction in the final model, with the rolling seven-day weighted wind direction (T_7w_) emerging as a predictor of capture success (F_1,139_ = 8.985, P<0.05). Successful surveys were associated with a nor’-nor’-east shift in wind direction when compared to surveys that were deemed unsuccessful (Figs 6 & 7). Upon further investigation, this trend was only significant for survey efforts at Haycock Island (F_1,54_ = 9.348, P<0.05) as, when the analysis was split by site, wind direction lost significance on all survey trips at Double Island (F_1,83_ = 1.958, P>0.05, Fig 7). For final best-fit model outputs see S3A & S5 Tables. Furthermore, unlike rainfall, this relationship between wind direction and animal presence was not robust across temporal timeframes. No pairwise relationships were established between wind and animal presence for shorter time periods with the significance of this environmental variable lost below T_7_ (S1 Table).

Despite the statistical associations, the overall explanatory power of the final, best-fit model was small with a marginalized R^2^ of 0.169. Therefore, Together rainfall (T_7_) and wind direction (T_7w_) accounted for only 16.9% of the observed variability in the presence/absence of *C*. *barnesi* medusa leaving 83.1% of the total variation unexplained.

### Environmental drivers of *Carukia barnesi* abundance

The optimal model for the environmental correlates of *Carukia barnesi* abundance (negative binomial, AIC = 192.0) included only measures of rainfall (T_0_) and weighted wind direction (T_7w_). Stinger season and site were included due to their significance in preliminary testing. Synonymous to part 1 of the hurdle model all other environmental predictors, including measures of sea surface temperature, salinity, tidal range, and moon phase (alongside the remaining temporal divisions of wind speed, and rainfall at T_0_) did not have a significant effect on model fit and were eliminated from the final selection. The top three ranking models, and their predictor variables, are reported in the S4B Table, and model output is reported in S6 Table.

**Fig 8:**
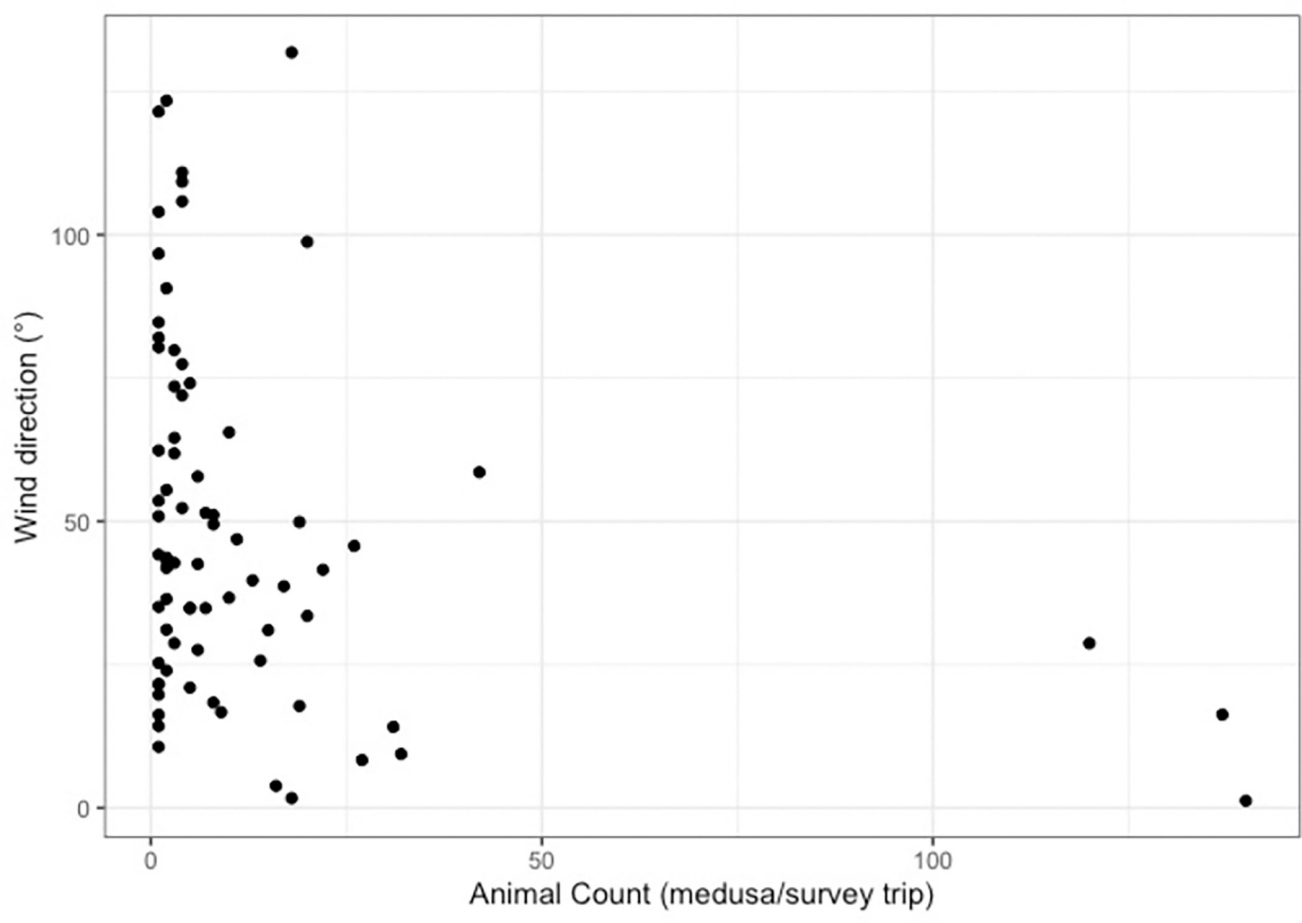
Wind direction and *Carukia barnesi* abundance. Mean weekly wind direction (T_7w_) associated with the number of jellyfish caught on survey trips. Points represent individual animal counts from singular survey trips. Data represent trips from all stinger seasons and sites. Data is zero truncated and graphed are only trips where jellyfish were caught.

**Fig 9:**
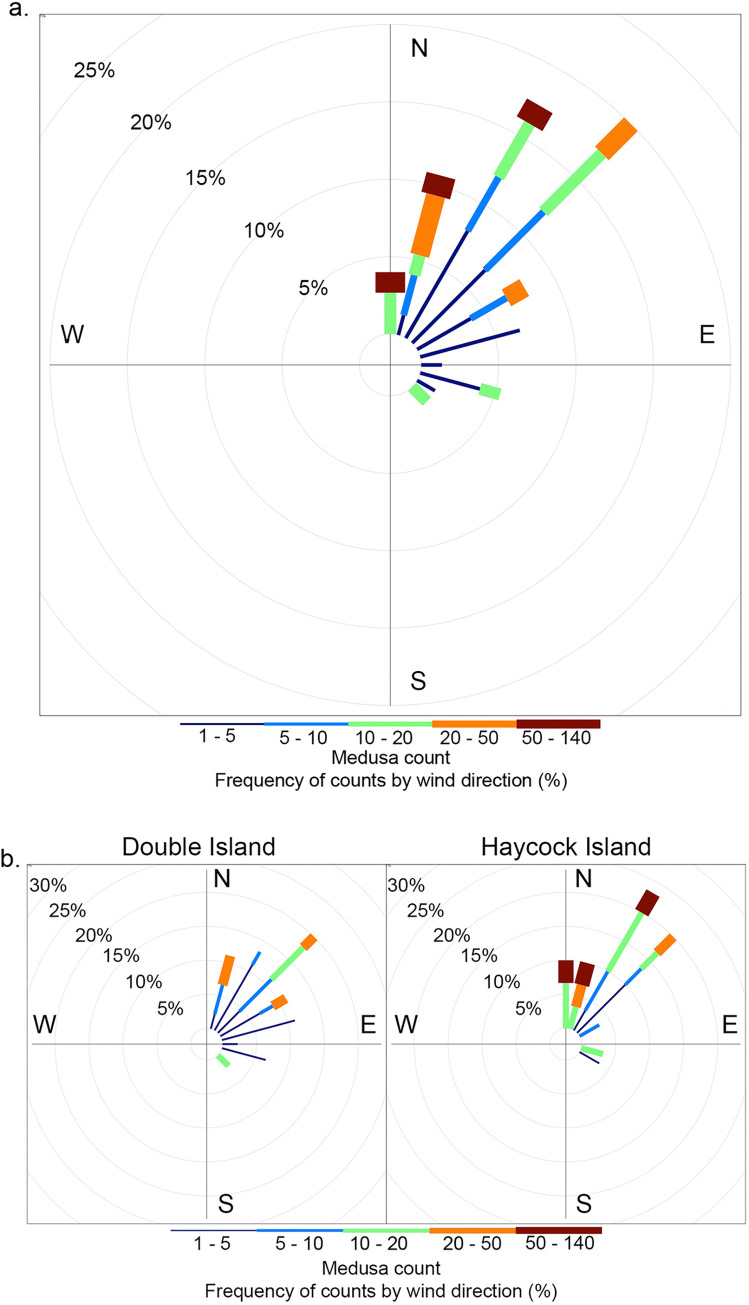
Categorical wind direction and *Carukia barnesi* abundance by site. Graphed is wind directions (T_7w_) associated with jellyfish medusa counts (a.) and data has been split by site (b.). Abundance has been categorized into 15-degree bins and colored by count. Data is zero truncated and graphed are only trips where jellyfish were caught.

The number of jellyfish caught on an individual sampling trip was significantly correlated with the mean wind direction seven days prior to catch (F_1,70_ = 6.866, P<0.01). High numbers of jellyfish (20–140 individuals per trip) were associated with nor’-nor’-east wind directions, originating between approximately 0 and 50°, whereas wind direction on trips associated with low jellyfish counts (0–10 individuals per trip) encompassed all directions (Figs 8 & 9). Survey trips for which catch tallies were in a medium (10–20 individuals per trip) range saw variation in wind direction from 0 to 180° but predominantly fell from 0 to 65° (Figs 8 & 9). This finding was consistent over site (Fig 9), however statistical significance depended on the temporal window with T_7_ being the only significant correlate (S2 Table).

While selected for in the final model no statistical relationship was established for trends in *C*. *barnesi* abundance and rainfall T_0_ (F_1,70_ = 0.461, P>0.05). Stinger season and site had a significant effect on *C*. barnesi abundance (F_1,70_ = 1.730, P<0.05, F_1,70_ = 2.550, P<0.05 year and site respectively).

In total the finalized model had a marginal R^2^ value of 0.209. Thus, the best-fit model, which included the environmental predictors of weighted wind direction and rainfall, explained approximately 20% of the total variation observed in trends of jellyfish abundance leaving 80% of the variation unexplained.

## Discussion

In this study we present a comprehensive database of *Carukia barnesi* occurrence and abundance records, spanning six stinger seasons, 159 individual surveys, and a combined capture of approximately 1000 medusae. Using direct capture data, we obtained insight pertaining to overarching patterns of medusa occurrence never-before reported in the literature, such as annual catch rates and records of medusa sizes, while correlative modeling facilitated exploration into the association between core environmental factors and patterns of occurrence and abundance for this species.

### Trends pertaining to annual abundance and medusa size

In this study, *C*. *barnesi* abundance was consistent across sites and stinger seasons as, despite the presence of three large catch days, overall variation in medusa abundance tallies was small ranging between 1.8 and 8.2 individuals per survey trip. Jellyfish, more commonly scyphozoan species, are well known for their capacity to occur in large numbers and singular ‘bloom’ events, tallying thousands of individuals, have been reported worldwide [16]. However, current literature supporting cubozoan abundance of this magnitude is sparse with only a small number of studies, reporting the occurrence of cubo-medusa in comparative numbers [14, 28, 37, 38]. While the presence of larger catch days in our study, where *C*. *barnesi* catch tallies were greater than 100 individuals, do demonstrate that large aggregations of *C*. *barnesi* are certainly possible the small frequency of these catch tallies over the experimental period, and consequently the consistency of ‘low catch tallies’, suggest these are uncommon. Alternatively, it is also possible that observed jellyfish abundance may reflect an uneven spatial distribution for this species and efforts should be made in the future to investigate this.

Throughout this study, we also observed an absence of the smallest size class of *C*.*barnesi* medusa (approximately 1 – 3mm) as no specimen was recorded having a niche bell width below 3mm (Fig 4). Historically, Double and Haycock Islands have been hypothesized to be potential ‘breeding grounds’ for *C*. *barnesi* [10] but, with the *in situ* locations of *C*. *barnesi* polyps still unknown, this is anecdotal and yet to be proven [7, 8, 39]. Instead, given the size distribution of captured medusa, what we propose is that it is possible the animals caught at these sites did not originate at these locations and were transported from elsewhere. While we acknowledge the absence of the smallest medusa size class could potentially be the result of sampling bias which would lend us to not detecting smaller jellyfish even if they were present, either due to the medusas being too small to detect in the water, having reduced swimming ability compared to larger individuals affecting their ability to circumvent currents and get to lights or the absence of phototactic behavior not attracting them to the sampling lights, we do have records of reliably catching smaller specimens of other cubo-medusa species, such as *Copula sivickisi*, alongside lab-based evidence of one-day-old medusa exhibiting strong propulsion and phototactic behaviors [39]. Regardless, this finding certainly warrants further exploration.

### Association between environmental correlates and patterns of medusa occurrence and abundance

The best-fit model-selection process identified measures of rainfall and weighted wind direction as the strongest environmental predictor variables for both animal presence and abundance. The likelihood of medusa presence significantly increased in instances of high rainfall and both the likelihood of medusa presence and abundance increased in nor’-nor’-easterly winds. While the multivariate models did perform better than their univariate equivalents, the overall explanatory power of both presence and abundance models was low. In total only between 16% and 20% of the overall variation in observed medusa presence and abundance, respectively, were explained by the final multi-variate best-fit models. Therefore, 80% and 84% of the total variation observed in occurrence and abundance patterns remains unexplained. What these findings suggest is that for the management of this medically important jellyfish species the assessed environmental variables are not particularly informative when used alone.

Furthermore, despite the significance of such variables in previous correlative jellyfish studies, neither model was improved by the inclusion of any of the remaining contestant variables such as measures of moon visibility, tide, sea surface temperature, river outflow, or wind speed. This is somewhat surprising, as associations have been established between these variables and other species of Cubozoan medusa [14, 22–25].

We believe the low predictive capacities identified in this study are a consequence of the current lack of ecological knowledge on this medically important species. Cubozoa is a class of jellyfish renowned for their sophisticated eye structures [40–42], capacity for advanced behaviors (such as diurnal migrations in the water column [43], sleeping [44], and active predatory behaviors [11]), and highly venomous sting [6, 8]. However, due to the logistically difficult nature of *Carukia barnesi* the ecological knowledge base surrounding this species, in particular, is still incredibly limited [7, 39, 45]. Thus while accurate associations and forecasting tools, based on environmental parameters, have been established for many species of scyphozoan jellyfish [15, 16, 32, 46] these studies have also benefitted from an established knowledge base surrounding the important aspects of life history, behavior, and ecology of these species (such as drivers of medusa metamorphosis and/or *in situ* polyp habitat and/or understanding of species behavior). For such studies, the availability of this ecological knowledge has enhanced the overall power of correlative models by aiding the establishment of causal linkages and mechanistic underpinnings for the observed occurrence trends. Therefore, while we acknowledge correlative models for *C*.*barnesi* may be enhanced by the addition of outstanding variables (e.g. information on local currents) until a good foundation of ecological understanding is established ‘top-down’ models will remail a case of ‘minimal understanding in, minimal understanding out.

### Implication for the current understanding of Irukandji occurrence patterns and recommendations for future research and management approaches

Historically, strong cultural/community associations have been drawn between the occurrence and abundance of Irukandji jellyfish and the long-term presence of north-easterly winds with many believing wind direction alone to be the sole driver of distribution patterns. In recent times, the reliance on windspeed for management decisions has been supported by scientific research correlating incidences of Irukandji envenomation (originating from hospital records) to the relaxation of south-easterly trade winds [13]. While the exact mechanism behind this trend remains unknown theories have been proposed by researchers suggesting these conditions lead to a shoreward movement of cooler oceanic water bodies carrying jellyfish to shore [13, 47]. Within the bounds of this study, we also found evidence that wind is a factor influencing *C*. *barnesi* occurrence and abundance, but that the predictive capacity for these environmental drivers is poor explaining less than 20% of the observed variability. In addition, these established associations were also highly site-specific, and the significance of trends was drastically affected by the temporal window in which they were observed. What these results suggest is that, within the bounds of study, there is not sufficient evidence to suggest wind direction alone drives the occurrence and abundance of the Irukandji jellyfish in Far North Queensland.

We believe this discrepancy may be connected to the nature of the response data (capture vs. sting records). To date, the majority of research on occurrence and abundance patterns of *C*. *barnesi* rely on incidental, or indirect, occurrence data in the form of hospital sting records [8, 13]. For indirect data such as this standardization is extremely difficult and consequently, the results are often subjected to inherent bias [8, 9]. In Far North Queensland the presence of north-easterly breezes is associated with high air temperatures and as a result, see a significant increase in the levels of beach occupancy [48]. However, studies such as that mentioned above are unable to account for the number of individuals in the water when a sting event occurs. Thus, potentially what is reported in these studies are not true trends of jellyfish occurrence and abundance but more an indicator of un-standardized sampling methods and how many individuals are in the water. In essence Nor’ easterly breezes lead to an increase in air temperature which increases beach occupancy inflating the number of individuals in the water and the probability of being stung. This phenomenon has been reported by other studies previously [8, 48] and while studies have attempted data standardization, via considering factors such as the availability of lifeguards on sting presentation [26], this is yet to be observed for historical *C*. *barnesi* sting records. While we acknowledge direct boat-based surveys, such as that used in this study, are certainly subject to limitations (for example a sampling bias towards safe conditions and the inherent inability to distinguish between ‘true’ and ‘false’ negatives) individual animal identification removes species ambiguity and data can easily be standardized for sampling effort. Until sting data can be accompanied by a direct animal identification and adjusted for the number of individuals in the water upon each sting event occurring, relying on this type of indirect data will produce anecdotal trends at best and the discrepancies between studies with direct and indirect observational data will remain.

In the future, research should target the association between patterns of *Carukia barnesi* occurrence and environmental drivers which were not possible in this study (for example variables such as turbidity, local currents, and water body movement). Studies should also consider the role animal behavior, such as co-occurrence with potential prey species and spawning cycles, may have on driving occurrence trends. However, in addition, we also believe research should also prioritize understanding foundational ecological relationships, for example–understanding the eco-physiological relationships (and limitations) between *C*. *barnesi* and core components of their environment, as these play a vital role in facilitating a mechanistic understanding of the occurrence and abundance patterns of this medically important organism.

## Conclusion

In conclusion, through a multi-season assessment of *C*. *barnesi* survey efforts, we have shown that patterns of medusa occurrence and abundance are variable in the nearshore waters off Cairns Queensland. While best-fit models showed a small amount of variation can be accounted for by long-term trends pertaining to environmental variables such as rainfall and wind direction, these associations demonstrated a lack of consistency across variation in temporal time frames and were dependent upon survey site location suggesting potential sampling bias. Ultimately, we demonstrated that the best-fit models had very low statistical inference power explaining between 16 and 20% of the overall variation in the data and leaving approximately 80% of all variation in medusa presence and abundance unexplained. Therefore, we believe, the occurrence and abundance patterns of *C*. *barnesi* medusa is not a simple linear relationship driven by a singular environmental predictor alone. Rather, we believe more research is needed on the ecology of this species before correlative relationships can be utilized in the predictive forecasting and management of this medically important species.

## Supporting information

S1 Fig Frequency of consecutive rain days. Figure shows the frequency of consecutive days of rainfall pooled for all seven stinger seasons spanning 2013/14 to 2018/19. Data is collated from October 1^st^ through April 30^th^ of each season representing 212 days per season. Days of rainfall were defined as having a daily total rainfall >0 mm and the initiation and completion of ‘consecutive rainfall day’ runs were defined by days recording 0 mm total precipitation.
(JPEG)

S2 Fig Rainfall and outflow collinearity plots. a—Rainfall vs. outflow, b—Weighted (W_Wd_) vs. non-weighted wind direction (NW_WD_). Temporal time frames are coded as T0 –day of catch, T3—day of catch and data for the previous two days, T5—day of catch and data for the previous four days, and T7—day of catch and data for the previous four days. Values represent Pearson’s correlation coefficients between environmental parameters. Colors represent the strength of association (low R^2^ value–yellow to strong R^2^ value–red).
(PNG)

S1 Table Summary of univariate logistic regression analysis on environmental predictor variables of *C*.*barnesi* survey success. Temporal time frames are coded as T_0_ –day of catch, T_3_ –day of catch and data for the previous two days, T_5_ –day of catch and data for the previous four days, and T_7_ –day of catch and data for the previous six days. Wind directions are coded as T_0w_ –weighted wind direction and T_0nw_−non-weighted wind direction.
(PDF)

S2 Table Summary of univariate negative binomial generalized linear analysis on environmental predictor variables of *C*.*barnesi* abundance. Temporal time frames are coded as T_0_ –day of catch, T_3_ –day of catch and data for the previous two days, T_5_ –day of catch and data for the previous four days, and T_7_ –day of catch and data for the previous six days. Wind directions are coded as T_0w_ –weighted wind direction and T_0nw_−non-weighted wind direction.
(PDF)

S3 Table Model selection tables for hurdle model parts one (a) and two (b). Displayed are the top three models, and associated parameters, for both parts of the hurdle model assessing environmental correlates of *C*. *barnesi* presence (a) and abundance (b). Listed are the associated parameters for each model, regression coefficients, degrees of freedom (df), log-likelihood, and Akaike’s Information Criterion (AIC). In total 23 individual parameters were assessed for model selection, originating from eight environmental variables and 1–5 time periods (for a full list see S1 Table).
(PDF)

S4 Table Summary of logistic regression model for the correlation of environmental variables on the presence/absence of jellyfish. Rainfall (T_7_) represents the mean hourly rainfall (mm/h) encompassing the day of sampling and six days prior, Wind direction (T_7w_) represents the mean weighted wind direction encompassing the day of sampling and six days prior.
(PDF)

S5 Table Summary of logistic regression model for the correlation of environmental variables on the presence/absence of jellyfish by site. Rainfall (T_7_) represents the mean hourly rainfall (mm/h) encompassing the day of sampling and six days prior, Wind direction (T_7w_) represents the mean weighted wind direction encompassing the day of sampling and six days prior Data has been split by site with the first table representing all surveys at Double island and the second at Haycock.
(PDF)

S6 Table Summary of negative binomial generalized linear model for the correlation of environmental variables on the number of jellyfish caught on sampling trips. Rainfall (T_0_) represents the mean hourly rainfall (mm/h) encompassing the day of sampling, Wind direction (T_7w_) day represents the mean weighted wind direction encompassing the day of sampling and six days prior.
(PDF)

S1 Data *Carukia barnesi* catch data. (XLSX)
