# Supplementary figures and images for: Environmental drivers of the occurrence and abundance of the Irukandji jellyfish (Carukia barnesi)
Source: PLoS One. 2022 Aug 4;17(8):e0272359. doi: 10.1371/journal.pone.0272359 (PMC9352007; doi:10.1371/journal.pone.0272359)

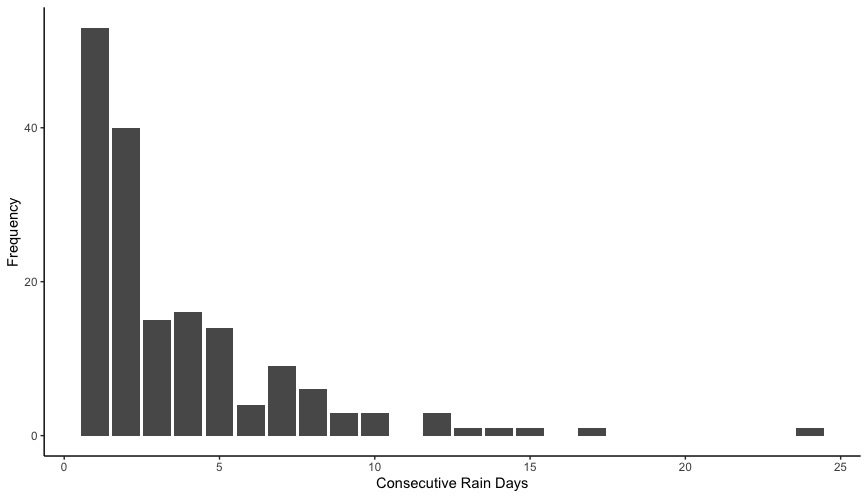

Supplement: S1 Fig — Figure shows the frequency of consecutive days of rainfall pooled for all seven stinger seasons spanning 2013/14 to 2018/19. Data is collated from October 1st through April 30th of each season representing 212 days per season. Days of rainfall were defined as having a daily total rainfall >0 mm and the initiation and completion of ‘consecutive rainfall day’ runs were defined by days recording 0 mm total precipitation. (JPEG) [file pone.0272359.s001.jpeg]

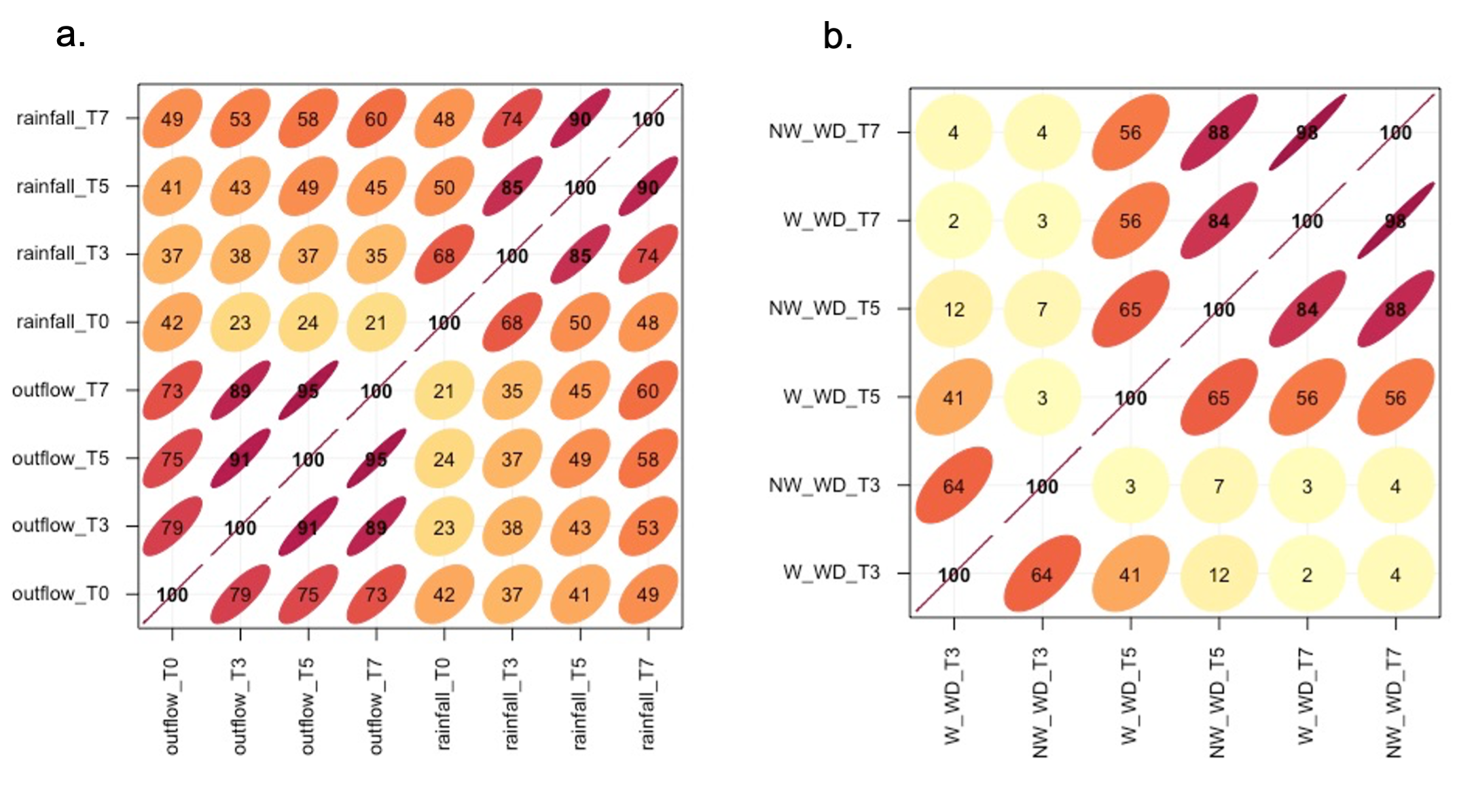

Supplement: S2 Fig — a—Rainfall vs. outflow, b—Weighted (W_Wd_) vs. non-weighted wind direction (NW_WD_). Temporal time frames are coded as T0 –day of catch, T3—day of catch and data for the previous two days, T5—day of catch and data for the previous four days, and T7—day of catch and data for the previous four days. Values represent Pearson’s correlation coefficients between environmental parameters. Colors represent the strength of association (low R2 value–yellow to strong R2 value–red). (PNG) [file pone.0272359.s002.png]
