# Supplementary material for: Environmental drivers of the occurrence and abundance of the Irukandji jellyfish (Carukia barnesi)
Source: PLoS One. 2022 Aug 4;17(8):e0272359. doi: 10.1371/journal.pone.0272359 (PMC9352007; doi:10.1371/journal.pone.0272359)
Supplement: S1 Table — Temporal time frames are coded as T0 –day of catch, T3 –day of catch and data for the previous two days, T5 –day of catch and data for the previous four days, and T7 –day of catch and data for the previous six days. Wind directions are coded as T0w –weighted wind direction and T0nw−non-weighted wind direction. (PDF) [file pone.0272359.s003.pdf]

Environmental variable	McFadden's P^2	Cox and Snell R^2	Naglerke's R^2	AIC	P value
Rainfall (T_0)	0.006	0.006	0.010	199.97	0.312
Rainfall (T_3)	0.045	0.064	0.086	191.53	**0.0067**
Rainfall (T_5)	0.058	0.077	0.103	189.57	**0.0025**
**Rainfall (T_7)**	**0.088**	**0.115**	**0.153**	**183.64**	**0.0002**
Outflow (T_0)	0.039	0.053	0.071	193.28	**0.0192**
Outflow (T_3)	0.075	0.098	0.131	186.26	**0.0027**
Outflow (T_5)	0.059	0.078	0.104	189.44	**0.0036**
**Outflow (T_7)**	**0.061**	**0.080**	**0.107**	**189.07**	**0.0026**
Wind direction (T_{0w})	0.011	0.014	0.019	198.97	0.1573
Wind direction (T_{3w})	0.020	0.027	0.037	197.09	0.817
Wind direction (T_{5w})	0.002	0.002	0.003	200.75	0.558
**Wind direction (T_{7w})**	**0.027**	**0.037**	**0.050**	**195.66**	**0.0233**
Wind direction (T_{3nw})	0.006	0.008	0.011	199.92	0.298
Wind direction (T_{5nw})	0.013	0.017	0.023	198.58	0.123
**Wind direction (T_{7nw})**	**0.021**	**0.029**	**0.039**	**196.85**	**0.0449**
Wind Speed (T_0)	0.001	0.001	0.001	200.96	0.753
Wind Speed (T_3)	**0.032**	**0.043**	**0.058**	**194.76**	**0.0196**
Wind Speed (T_5)	**0.054**	**0.071**	**0.095**	**190.51**	**0.0028**
**Wind Speed (T_7)**	**0.059**	**0.078**	**0.105**	**189.39**	**0.0017**
Sea surface temperature (T_0)	**0.028**	**0.038**	**0.051**	**195.45**	**0.0238**
Moon phase (T_0)	0.009	0.013	0.017	199.23	0.1781
Tide range (T_0)	**0.027**	**0.037**	**0.049**	**195.27**	**0.0237**

Listed are the associated parameters for each model goodness of fit - McFadden's P^2, Cox and Snell R^2, Naglerke's R^2), assessment criteria (Aikaike's information criterion) and significance values. Significant values ($P<0.05$) are bolded.
