# Supplementary material for: Environmental drivers of the occurrence and abundance of the Irukandji jellyfish (Carukia barnesi)
Source: PLoS One. 2022 Aug 4;17(8):e0272359. doi: 10.1371/journal.pone.0272359 (PMC9352007; doi:10.1371/journal.pone.0272359)
Supplement: S2 Table — Temporal time frames are coded as T0 –day of catch, T3 –day of catch and data for the previous two days, T5 –day of catch and data for the previous four days, and T7 –day of catch and data for the previous six days. Wind directions are coded as T0w –weighted wind direction and T0nw−non-weighted wind direction. (PDF) [file pone.0272359.s004.pdf]

**Environmental variable**	**McFadden's P^2**	**Cox and Snell R^2**	**Naglerke's R^2**	**AIC**	**P value**
Rainfall (T_0)	0.00336	0.02219	0.02222	505.58	0.312
Rainfall (T_3)	0.00272	0.01801	0.01803	505.90	0.325
Rainfall (T_5)	0.00130	0.00867	0.00868	506.61	0.489
Rainfall (T_7)	0.00207	0.01373	0.01375	506.23	0.346
Outflow (T_0)	0.00340	0.02247	0.02250	505.56	0.177
Outflow (T_3)	0.00424	0.02797	0.02800	505.14	0.146
Outflow (T_5)	0.00405	0.02673	0.02676	505.24	0.156
**Outflow (T_7)**	**0.00809**	**0.05264**	**0.05271**	**503.21**	**0.036**
Wind direction (T_{0w})	0.0000 2	0.00014	0.00014	507.26	0.926
Wind direction (T_{3w})	0.00397	0.02620	0.02623	505.28	0.263
Wind direction (T_{5w})	0.00590	0.03866	0.03871	508.05	0.931
**Wind direction (T_{7w})**	**0.02234**	**0.13872**	**0.13890**	**496.07**	**0.002**
Wind direction (T_{3nw})	0.00317	0.02099	0.02102	505.68	0.248
Wind direction (T_{5nw})	0.01707	0.10779	0.10793	498.71	**0.009**
Wind direction (T_{7nw})	0.01713	0.10819	0.10833	498.68	**0.008**
Wind Speed (T_0)	0.00124	0.00823	0.00824	506.65	0.441
Wind Speed (T_3)	0.00036	0.00238	0.00239	507.09	0.686
Wind Speed (T_5)	0.00009	0.00059	0.00059	507.22	0.838
Wind Speed (T_7)	0.00009	0.00006	0.00006	507.26	0.949
Sea surface temperature (T_0)	0.00307	0.02032	0.02035	505.73	0.155
**Moon phase (T_0)**	**0.01448**	**0.09224**	**0.09235**	**500.01**	**0.004**
Tide range (T_0)	0.00033	0.00221	0.00221	507.10	0.705

Listed are the associated parameters for each model goodness of fit - McFadden's P^2, Cox and Snell R^2, Naglerke's R^2), assessment criteria (Aikaike's information criterion) and significance values. Significant values ($P<0.05$) are bolded.
