# Supplementary material for: Environmental drivers of the occurrence and abundance of the Irukandji jellyfish (Carukia barnesi)
Source: PLoS One. 2022 Aug 4;17(8):e0272359. doi: 10.1371/journal.pone.0272359 (PMC9352007; doi:10.1371/journal.pone.0272359)
Supplement: S3 Table — Model selection tables for hurdle model parts one (a) and two (b). Displayed are the top three models, and associated parameters, for both parts of the hurdle model assessing environmental correlates of C. barnesi presence (a) and abundance (b). Listed are the associated parameters for each model, regression coefficients, degrees of freedom (df), log-likelihood, and Akaike’s Information Criterion (AIC). In total 23 individual parameters were assessed for model selection, originating from eight environmental variables and 1–5 time periods (for a full list see S1 Table). (PDF) [file pone.0272359.s005.pdf]

(a) **Hurdle model part one *C. barnesi* presence/absence – logistic regression.**

Model ranking	Intercept	Sea surface temperature (T_0)	Tide magnitude (T_0)	Mean rainfall (T_7)	Wind direction (T_{7w})	McFadden's P^2	Cox and Snell R^2	Naglerke's R^2	df	Log likelihood	AIC
1	0.18920	NA	NA	0.10270	-0.018080	0.135	0.170	0.227	3	-92.903	176.6
2	-5.6350	0.1966	NA	0.09858	-0.017390	0.144	0.180	0.240	4	-92.110	177.0
3	0.72380	NA	-0.367204	0.09624	-0.018120	0.140	0.180	0.235	4	-92.687	177.7

(b) Hurdle model part two *C. barnesi* abundance – negative binomial regression.

Model ranking	Intercept	Site	Season	Moon illumination (T_0)	Mean rainfall (T_0)	Wind direction (T_{7w})	McFadden's P^2	Cox and Snell R^2	Naglerke's R^2	df	Log likelihood	AIC
1	3.051	+	+	NA	0.009979	-0.009180	0.0879	0.4441	0.4446	9	-228.615	478.0
2	3.039	+	+	NA	NA	-0.008432	0.0811	0.4184	0.4190	8	-230.307	478.8
3	2.991	+	+	0.1975000	0.009385	-0.009575	0.0883	0.4460	0.4465	10	-228.497	480.4
