# Supplementary material for: Environmental drivers of the occurrence and abundance of the Irukandji jellyfish (Carukia barnesi)
Source: PLoS One. 2022 Aug 4;17(8):e0272359. doi: 10.1371/journal.pone.0272359 (PMC9352007; doi:10.1371/journal.pone.0272359)
Supplement: S4 Table — Rainfall (T7) represents the mean hourly rainfall (mm/h) encompassing the day of sampling and six days prior, Wind direction (T7w) represents the mean weighted wind direction encompassing the day of sampling and six days prior. (PDF) [file pone.0272359.s006.pdf]

**Coefficients**	**Estimate**	**Sd. Error**	**Z value**	**P-value**
Intercept	0.189161	0.369586	0.512	0.60878
Rainfall (T_7)	0.102750	0.025921	3.964	7.37e-05
Wind direction (T_{7w})	-0.018082	0.006215	-2.909	0.00362
