# Supplementary material for: Environmental drivers of the occurrence and abundance of the Irukandji jellyfish (Carukia barnesi)
Source: PLoS One. 2022 Aug 4;17(8):e0272359. doi: 10.1371/journal.pone.0272359 (PMC9352007; doi:10.1371/journal.pone.0272359)
Supplement: S5 Table — Rainfall (T7) represents the mean hourly rainfall (mm/h) encompassing the day of sampling and six days prior, Wind direction (T7w) represents the mean weighted wind direction encompassing the day of sampling and six days prior Data has been split by site with the first table representing all surveys at Double island and the second at Haycock. (PDF) [file pone.0272359.s007.pdf]

**Coefficients (Double)**	**Estimate**	**Sd. Error**	**Z value**	**P-value**
Intercept	-0.263892	0.505875	-0.522	0.601911
Rainfall (T_7)	0.107441	0.031810	3.378	**0.000731**
Wind direction (T_{7w})	-0.011101	0.007726	-1.437	0.150733
**Coefficients (Haycock)**	**Estimate**	**Sd. Error**	**Z value**	**P-value**
Intercept	0.89624	0.59839	1.498	0.13420
Rainfall (T_7)	0.08791	0.04429	1.985	**0.04718**
Wind direction (T_{7w})	-0.03160	0.01175	-2.689	**0.00717**
