# Supplementary material for: Environmental drivers of the occurrence and abundance of the Irukandji jellyfish (Carukia barnesi)
Source: PLoS One. 2022 Aug 4;17(8):e0272359. doi: 10.1371/journal.pone.0272359 (PMC9352007; doi:10.1371/journal.pone.0272359)
Supplement: S6 Table — Rainfall (T0) represents the mean hourly rainfall (mm/h) encompassing the day of sampling, Wind direction (T7w) day represents the mean weighted wind direction encompassing the day of sampling and six days prior. (PDF) [file pone.0272359.s008.pdf]

Coefficients	Estimate	Sd. Error	Z value	P-value
Intercept	6.394923	1.307618	4.891	**1.01e-06**
Rainfall (T_0)	0.012779	0.007314	1.747	00.08060
Wind direction (T_{7w})	-0.013382	00.004380	-3.055	**0.00225**
Season	-0.245012	0.080405	-3.047	**0.00231**
Site	0.727070	0.273352	2.660	**0.00782**
